# Contamination Level, Ecological Risk, and Source Identification of Heavy Metals in the Hyporheic Zone of the Weihe River, China

**DOI:** 10.3390/ijerph17031070

**Published:** 2020-02-07

**Authors:** Muhammad Irfan Ahamad, Jinxi Song, Haotian Sun, Xinxin Wang, Muhammad Sajid Mehmood, Muhammad Sajid, Ping Su, Asif Jamal Khan

**Affiliations:** 1 Shaanxi Key Laboratory of Earth Surface System and Environmental Carrying Capacity, College of Urban and Environmental Sciences, Northwest University, Xi’an 710127, China; irfan@stumail.nwu.edu.cn (M.I.A.); sunhaotian@nwu.edu.cn (H.S.); xinxinwang@stumail.nwu.edu.cn (X.W.); m.sajid.mehmood@hotmail.com (M.S.M.); pingsu@stumail.nwu.edu.cn (P.S.); asifj_khan@yahoo.com (A.J.K.); 2 Institute of Qinling Mountains, Northwest University, Xi’an 710127, China; 3 State Key Laboratory of Soil Erosion and Dryland Farming on the Loess Plateau, Institute of Soil and Water Conservation, Chinese Academy of Sciences and Ministry of Water Resources, Yangling 712100, China; 4 Key Laboratory for Industrial Biocatalysis, Ministry of Education of China, Institute of Applied Chemistry, Department of Chemical Engineering, Tsinghua University, Beijing 100084, China; wang-li17@mails.tsinghua.edu.cn; 5 Department of Chemical Engineering, University of Gujrat, Gujrat 50700, Pakistan

**Keywords:** sediments, heavy metals, geo-accumulation index, potential ecological risk, Weihe River

## Abstract

The sediment pollution caused by different metals has attracted a great deal of attention because of the toxicity, persistence, and bio-accumulation. This study focuses on heavy metals in the hyporheic sediment of the Weihe River, China. Contamination levels of metals were examined by using “geo-accumulation index, enrichment factor, and contamination factor” while ecological risk of metals were determined by “potential ecological risk and risk index”. The pollutant accumulation of metals ranked as follows: “manganese (Mn) > chromium (Cr) > zinc (Zn) > copper (Cu) > nickel (Ni) > arsenic (As) > lead (Pb)”. The geo-accumulation index identified arsenic as class 1 (uncontaminated to moderate contamination), whereas Cu, Cr, Ni, Zn, Pb, and Mn were classified as class 0 (uncontaminated). According to the enrichment factor, arsenic originated through anthropogenic activities and Cr, Ni, Cu, Zn, and Pb were mainly controlled by natural sources. The contamination factor elucidated that sediments were moderately polluted by (As, Cr, Cu, Zn, Mn, and Pb), whereas Ni slightly contaminated the sediments of the Weihe River. All metals posed a low ecological risk in the study area. The risk index revealed that contribution of arsenic (53.43 %) was higher than half of the total risk.

## 1. Introduction

The hyporheic zone is regarded as the immersed region underneath the riverbed, where the fraternization of ground and surface water typically happens [1]. It is a dynamic region that acts as a transitional zone for water exchange, material cycles, solute transport, and other ecological service functions [2]. The rivers perform multiple functions, including aquaculture, water transportation, irrigation, as well as provide domestic water. According to different policies and scientific objectives, several ecological functions of the river have been evaluated and studied, which include quality of water [3], hydrological processes [4], animal population dynamics [5], quality of sediments [6], and composition of vegetation [7]. Among these factors, the quality of the sediments has attracted particular attention, since the sediment not only acts as a reservoir for pollutants but also interacts with different factors [8]. For example, the sediment quality is related to hydrological connection, vegetation characteristics, quality of water, industrial material and process, land use, and mineral type [9]. As a result of industrial development, the water environment is increasingly exposed to metal pollution, due to their persistence, ability to incorporate within the food chain, and environmental bioaccumulation [10,11]. Due to hydrolysis, co-precipitation, and adsorption, heavy metals are predominantly deposited in the sediment, with only a few dissolved in water [12].

Pollution caused by heavy metals is regarded as a severe risk to the river environment because of its chronic nature, toxicity, non-biodegradability, as well as bioaccumulation [13]. Heavy metal in polluted habitats can accumulate in river flora and fauna, which may enter into the food chain and create health problems [14]. Sediments are ecologically valuable constituents of the river environment [15]. Sediments acting as a carrier are also the secondary sources of pollutants in the river environment [16]. Therefore, the evaluation of the river sediments is a valuable approach to assess metal pollution in a given area [17].

Heavy metals have attracted researchers’ attention because of their toxicity, bioaccumulation, non-degradability, and enormous sources, together with their persistence in the aquatic environment [18]. After being released, heavy metals may be distributed in various components of the river environment [19]. As a result, simply a small quantity of heavy metals stay inside those water columns, and the maximum amount accumulated within the sediment [20]. Particularly, metals are combined with sediments by numerous mechanisms, including co-precipitation, surface adsorption particle, ion exchange, as well as complexation upon organic matters [21,22].

Within sediments, metals originate either from natural sources (for example atmospheric precipitation, ore deposits, geological weathering, disintegration of parent rocks because of storms, wind bioturbation, and waves), or by anthropogenic activities (for example mining, transportation industrial emission, smelting, fuel production, electroplating, sludge dumping, power transmission, dust, intensive urban and agricultural activities, and wastewater irrigation) [23,24,25]. Within the soil ecological community, the toxicity along with the mobility of heavy metals depends on different factors, including metal binding condition, chemical type, total accumulation, and properties of metals [26].

An enormous portion of heavy metals is directed toward aquatic surroundings and accumulated in the sediments, which can (a) contaminate water, causing the death of a regional aquatic population and accumulate in plants by means of irrigation [27]; (b) release into water by sediment re-suspension, desorption and adsorption reactions, oxidation and reduction reaction, together with degradation of the organisms [28,29].

Heavy metals are categorized as essential and nonessential metals. Essential metals occur naturally, while the nonessential metals, having no positive effect, are considered hazardous even in low quantity [30]. However, excessive use of essential metals has been linked to cellular and systemic disorders [31]. Further, in the long term, the accumulation of these metals in soil can lead to the deterioration of agricultural land, eutrophication, and the absorption of toxic substances [32]. In the last few years, natural sources and anthropogenic activities have contributed to an increasing level of heavy metals. Therefore, an evaluation is necessary to measure heavy metals concentration and understand the soil quality. There is a demanding need to carry out scientific research in terms of heavy metal pollution.

Our work addresses the distribution, contamination levels, metal sources, and heavy metal ecological risks. In this study, samples have been taken from several selected locations from the research area. This study aims to (1) evaluate the heavy metals “Arsenic (As), Chromium (Cr), Copper (Cu), Nickel (Ni), Lead (Pb), Zinc (Zn), and Manganese (Mn)”; (2) assess different levels of pollution, which include “geo-accumulation index, enrichment factor and contamination factor”; (3) assess the “potential ecological risk and ecological risk index” of metals in sediment; (4) evaluate the correlation and source identification of heavy metals.

## 2. Materials and Methods

### 2.1. Description of Study Area

**Figure 1:**
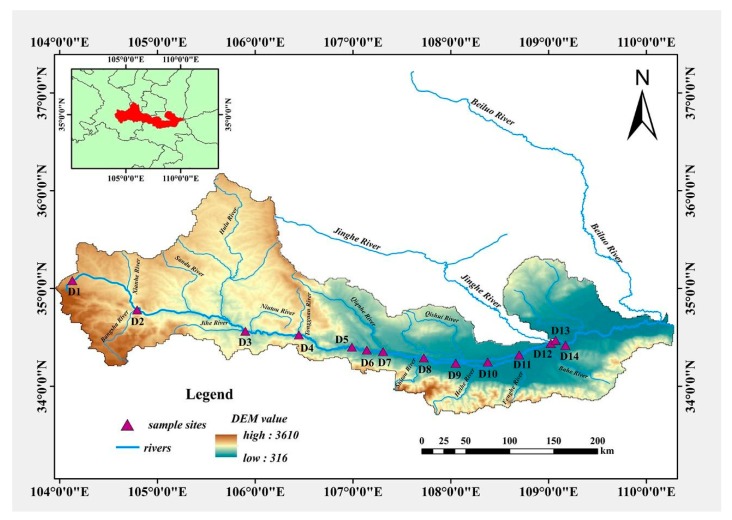
Sampling sites and study area maps.

The Weihe River is the biggest tributary of the Yellow River, having a length of almost 818 Km and flowing into the Yellow River in the Shaanxi Province (Figure 1). As the “mother river” of the Guanzhong region, the Weihe River is the primary agricultural and industrial region in northwest China [33]. The Weihe River is the main water supplier in central China and it covers an area of almost $6.67\;\times \;10^{4\;}{\mathrm{km}}^{2}$ in Shaanxi province [1,34]. In the province, average annual river flow and sediment yield are 103.7 × 10^8^ m^3^ and 5.8 × 10^8^ t, respectively, which accounts for one-third of the sediment load of the Yellow River. Loess Plateau, Qinling Mountains, and the Guanzhong Plain are three topographic classes covering the river basin.

The Weihe River is under a typical continental monsoon climate with dry, cold winters as well as rainy, hot summers, with heavy rainfall from June to October. Moreover, the river basin is predominantly covered with loess because of severe sediment transport and erosion [1].

### 2.2. Sampling

Sediment samples were collected from different sites of the Weihe River having the same properties. We selected fourteen sites from the mainstream of the river, which included upstream (D1–D6), middle stream (D7–D11), and downstream (D12–D14). These sites covered the overall mainstream and represented the pollution situation. Each sample consisted of triplicate. A “Global Positioning System” was used throughout the sampling to locate the exact location of the sampling sites. A thin-walled transparent poly-carbon tube with an opening at both ends was put into the riverbed sediment for samples. A piston column sampler was used where needed. The samples were preserved in polyethylene sampling bags and shifted back to a research laboratory within 12 h for further analysis.

### 2.3. Quality Control and Quality Assurance

Throughout the study, we assured quality control and quality assurance. Accuracy and precision were verified using reference materials sediment-certified samples “GBW07311 (GSD-11) and GBW07366 (GSD-23) of the National Center of China”. Instruments were calibrated before every analysis. The blanks in every set had been tested in duplicates using the same techniques. The results presented are the average values of duplicated analysis. For repeat tests, samples were selected randomly. All glasses, plastics, and quartz were cleaned in 10% HNO_3_, and ultrapure water (18.25 M ohm cm^-1^) was used to rinse every time.

### 2.4. Analysis of Samples

Stone and plant pieces were separated from the sediment samples. The sediments were grinded with agate mortar, powdered, and passed through 200 mesh nylon sieves, and finally stored in glass bottles that had been washed by nitric acid and water (3:1). For the evaluation of metal contents, concentrated HNO_3_, HF, and H_2_O_2_ were used to digest sediment samples [35]. A high temperature and high-pressure digester and electro thermal plate digestion were used during the digestion process. The filtration of the digested solution was done by using a filter (0.45 μm) “Xinya Purify Device Company, Shanghai, China”. Next, Cu, Cr, Ni, Zn, and Pb were assessed by an “Inductively coupled plasma mass spectrometer” (X series 2) manufactured by American Thermoelectric. Moreover, Fe and Mn were assessed by the “Inductively coupled plasma emission spectrometer” (Icap7400) manufactured by American Thermoelectric and Arsenic, by a two-channel atomic fluorescence photometer (AFS2000) manufactured by Beijing Haiguang.

### 2.5. Calculation of Pollution Levels

Various techniques had been used to estimate metal pollution in sediments. In this research, pollution levels, specifically “geo-accumulation index, enrichment factor, and contamination factor,” have been determined to evaluate different pollution levels. The selection of background value is the essential parameter to interpret useful geochemical data; average crustal value has been used as a background value by various researchers [24,36,37,38]. In this study, the average crustal value of metals presented by “Turekian and Wedepohl” has been used as the background value (Table S1) [39].

#### 2.5.1. Geo-Accumulation Index (Igeo)

Muller presented the concept of the Igeo, which is used to assess different pollution levels in soil and sediments. [40]. Igeo is measured by Equation (1).

(1) $$\mathrm{Igeo}=log2\frac{C_{n}}{1.5*B_{n}}$$

In Equation (1), $C_{n}$ shows measured metal contents in samples, $B_{n}$ symbolize reference or background values of metal, 1.5 is a factor that is used to calculate possible changes in background value. By using the average shale value, the geo-accumulation index was calculated [41]. Igeo comprises seven different classes (Table 1) [6].

#### 2.5.2. Enrichment Factor (EF)

EF is an excellent technique to calculate the proportion of pollutants in sediments [42]. EF for every metal was calculated to estimate how much metals are originated from anthropogenic activities in sediments [43,44]. EF is mostly used to differentiate the source of metals, which can be natural or anthropogenic [45]. It involves the stabilization of sediments relative to reference elements, for example, scandium (Sc), titanium (Ti), and manganese (Mn) [46], iron (Fe), and aluminum (Al) [47]. Anthropogenic metal enrichment was measured by using manganese (Mn), a reference element as most symbolized by Loska [48]. The equation given below is used to estimate the EF.

(2) $$EF=\frac{\left(\frac{C_{n}}{C_{Mn}}\right)\;sample}{\left(\frac{C_{n}}{C_{Mn}}\right)\;Background\;value}$$

Enrichment factor is the proportion between the desired sample to the world average background value from “Turekian and Wedepohl” [39]. Various contamination classes are determined with the help of enrichment factors (Table 1) [49,50].

#### 2.5.3. Contamination Factor (CF)

CF is deemed as a useful tool to monitor contamination in sediments over time. It is the ratio of every metal in the present sample to the background values in the same metal [36].

(3) $$CF=\frac{C_{heavy\;metal}}{C_{background}}$$

The contamination degrees can be categorized according to their values from 1 to 6 “if CF<1, low pollution; 1<CF<3, moderate pollution; 3<CF<6, considerable pollution; CF>6, very high pollution” [51]

### 2.6. Potential Ecological Risk and Risk Index

Hakanson presented a technique that was used to measure different levels of ecological risk in river sediment [52,53]. This approach assesses different levels of pollution in sediment while combining the environmental and ecological risks with toxicology, to assess potential risks and levels of metal pollution index [54].

(4) $$E_{r}^{i}=T_{r}^{i}*\frac{C_{i}}{C_{0}}$$

(5) $$RI=\overset{n}{\underset{i=1}{\sum }}T_{r}^{i}*\frac{C_{i}}{C_{O}}$$

where $C_{i}\;$represents the concentration in sediment i; *C_o_* shows the concentration in reference; $T_{r}^{i}$ describes the toxicity factor, which was already described as: As=10, Cu=Pb=Ni=5, Zn=Mn=1, Cr=2 [52,53]; $E_{r}^{i}$ represents the “ecological risk,” and RI denotes the overall “risk index” of metal. Different levels of risk index are presented in Table 2.

### 2.7. Statistical Analysis

Information about the source of contaminants was attained through the details of sediment conditions and statistical analysis [55]. Pearson’s correlation was used to examine the correlation between the concentrations of different metals. The PCA was used to extract a subset of the factors from the original variable [52,56]. Kaiser-Meyer-Olkin, the Bartlett sphericity test, as well as the covariance matrix were used on the base of eigenvalue to validate the PCA [8,57]. Varimax rotation was selected to measure metals and the rate of contribution for eigenvalues > 1 in principal components [53].

## 3. Results

### 3.1. Estimation of Heavy Metals in the Sediments of the Weihe River

The average concentration of As, Cr, Ni, Cu, Zn, Pb, and Mn were 29.16, 109.98, 41.47, 52.37, 103.47, 24.44, 888.29 mg/kg respectively. The concentration of Mn was higher than other heavy metals, whereas low concentrations of Pb were detected. The concentrations of Cr ranked second highest in sediment samples. The average value of As was 29.16 mg/kg, which is greater than the average shale value (13 mg/kg). Meanwhile, Pb average concentration was 24.44 mg/kg. The detailed concentrations of metals obtained from sediments are presented in Table 3.

In the case of Ni, the concentration was less than the average value of the shale at all sample locations. The copper concentrations in the D1 sediment was 69.34 mg/kg, probably from the industrial and urban waste [58]. Metals concentrations in the Weihe River were therefore ranked in descending order: Mn>Cr>Zn>Cu>Ni>As>Pb (Figure S1).

A comparison of heavy metal concentration with the data of other rivers from literature are listed in Table 4. The Chenab River, Pakistan [59], had low heavy metal concentration, and Axios River, Greece [60], River Po, Italy [61], and Tees River, UK [62], had high concentration as compared with the Weihe River. Moreover, the concentration of metals in the Weihe River sediments was almost at the center of the surveyed rivers in China [63,64,65,66]. The results showed that the concentrations of Cu, As, Cr, and Ni in the sediments were approximately equal to Zijiang River, Hunan and Yellow River, China. In Jialu River, China, except for As, metal concentrations were almost the same. Cu, Cr, Zn, and Pb are rather low when compared with the Yangtze River, China. In the Weihe River, the concentrations of As, Cr, and Ni were more significant than those in Luanhe River, China.

### 3.2. Contamination Level

#### 3.2.1. Geo-Accumulation Index (Igeo)

**Figure 2:**
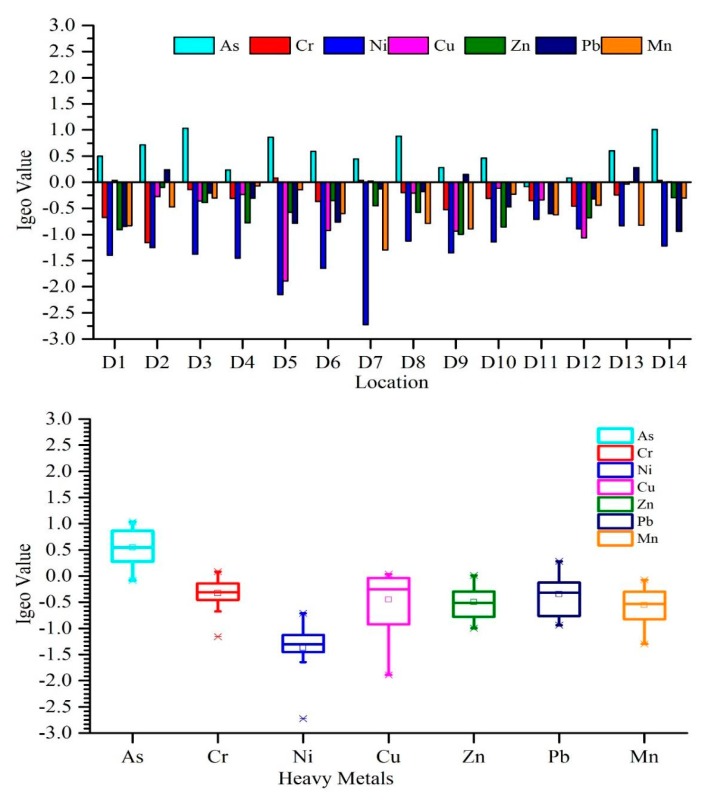
Variation in the (Igeo) values at different study sites in the Weihe River.

The Igeo was used to explain the quality of sediment [73]. The values of Igeo from all sampling sites are presented in Figure 2. Igeo values indicated that the Weihe River was not contaminated by Mn and Ni (Igeo<0), “unpolluted to moderately polluted” by Cr, Cu, Zn, and Pb (Igeo<1), and moderately contaminated by As (Igeo<2). The average Igeo values were ranked as: As>Cr>Pb>Cu>Zn>Mn>Ni (Figure 2).

#### 3.2.2. Enrichment Factor (EF)

**Figure 3:**
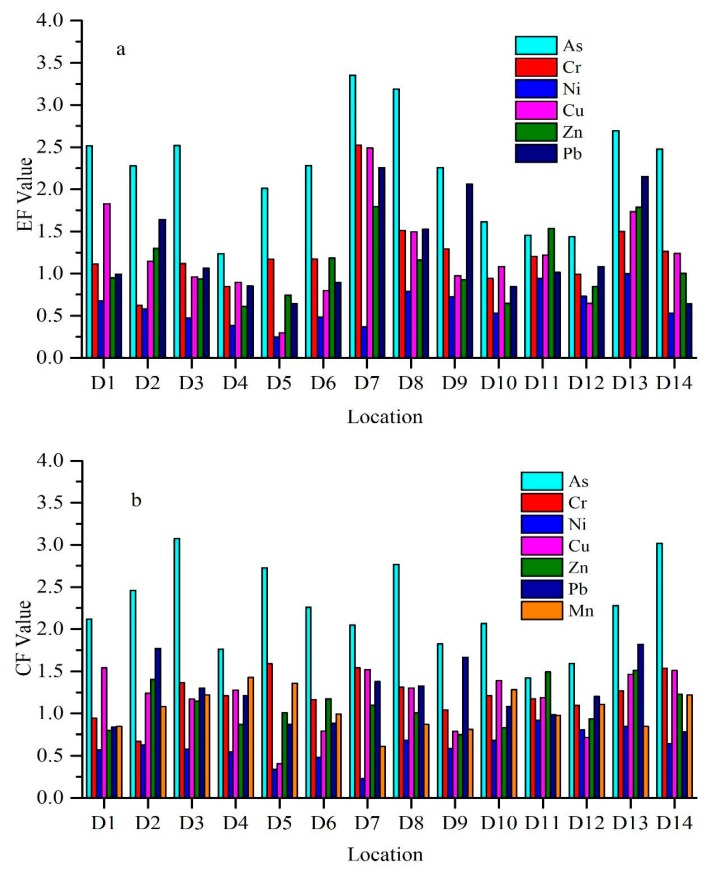
Variation in (**a**) enrichment factor (EF), (**b**) contamination factor (CF) from all study stations in the Weihe River.

EF is a common normalization procedure for classifying metal particles related to sediments. Generally, the average EF value of all the metals studied showed their enrichment in the Weihe River sediments (Figure 3). The highest EF value was found at site D7 (3.35) for As, which indicated deficiency to moderate enrichment. The lowest EF value was found at site D5 (0.25) for Ni, with no enrichment. EF values for As at all sites, except D4, D10, D11, and D12, Cr, and Cu at site D7, and Pb at D7 and D13 are greater than 2 in sediments, which shows deficiency to moderate enrichment. The highest EF value was identified in As, while the minimum in Ni. Pb has the second-highest EF value.

#### 3.2.3. Contamination Factor (CF)

Average CF concentrations of As, Cr, Ni, Cu, Zn, P, and Mn were 2.24, 1.22, 0.61, 1.16, 1.09, 1.22, and 1.05, respectively. CF value ranges for Cr, Ni, Cu, Zn, Pb, and Mn were 0.67 to 1.59, 0.23 to 0.92, 0.41 to 1.54, 0.75 to 1.51, 0.78 to 1.82, and 0.61 to 1.41, respectively. Among all heavy metals, Ni had the lowest value at site D7 (0.227), and As the highest value at site D3 (3.07). Average CF values for all metals were ordered as follows: As>Pb>Cr>Cu>Zn>Mn>Ni (Table S2).

### 3.3. Potential Ecological Risk and Risk Index

**Figure 4:**
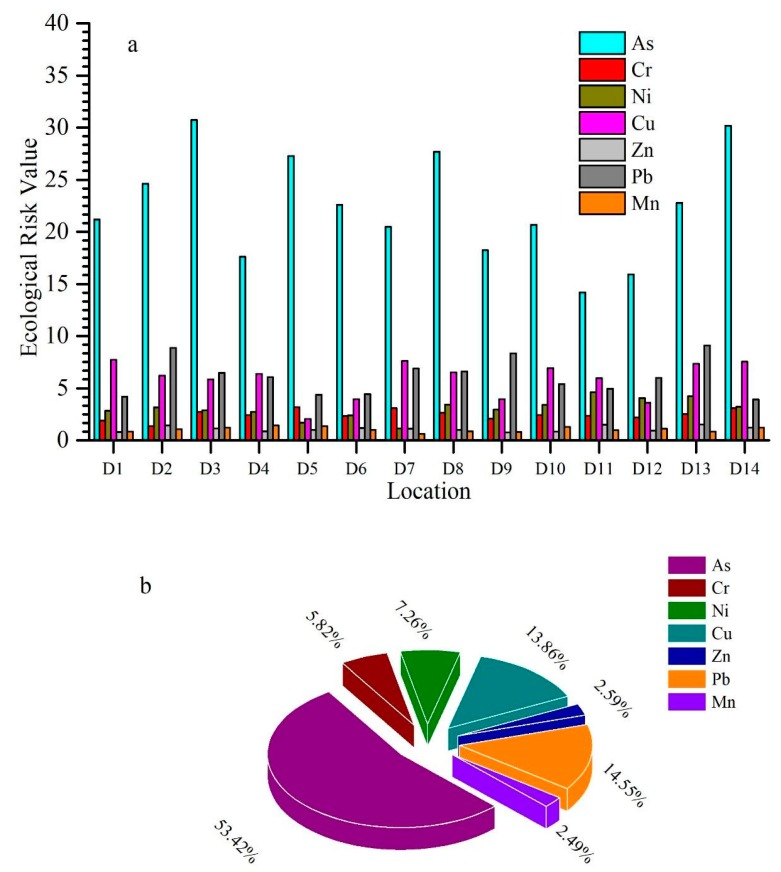
(**a**) Variation in the ecological risk of metals, (**b**) average percentage of individual metal in risk index.

Potential ecological risk of single metal, as well as the risk index (RI) of combined metals were measured; findings are shown in Figure 4. The Håkanson Index furnished quantitative techniques for isolating the potential hazard directly. However, its drawbacks are high stage subjectivity and ignorance of combined antagonism, or the weighting role of many heavy metals. In general, a single metal pollution index and risk index produce different results [74].

Based on the individual ecological risk, the highest ecological risk for arsenic was observed at D3 (30.71), and lowest at D11 (14.18) for chromium, the highest ecological risk at D5 (3.18), for Ni at D11 (4.59), for copper at D1 (7.71), for zinc at D13 (1.51), for lead at D13 (9.10), and Mn at D4 (1.43). The potential ecological risk (PER) for individual metal shows that the degree of metal contamination is in the following sequence: As > Pb > Cu > Ni > Cr > Zn > Mn (Figure 4).

The maximum risk index found at D3 (51.02), and the minimum risk index was at D12 (33.76). For the contribution of metals in potential risk index (RI), As was regarded as the key potential ecological risk factor, with contributions of 53.43 %, and other metals Cr, Ni, Cu, Zn, Pb, and Mn contributed 5.82%, 7.26%, 13.86%, 2.59%, 14.55%, and 2.49%, respectively. The risk index followed the order of D3 > D14 > D8 > D13 > D2 > D10 > D7 > D5 > D1 > D6 > D4 > D9 > D11 > D12 (Table S2).

### 3.4. Correlation among Heavy Metals

The correlation can identify the source and movement of metals among heavy metals [55,75]. Many metal pairs had positive correlations (P < 0.01): As-Cu (0.092), As-Mn (0.214), Ni-Zn (0.361), Cu-Zn (0.203), and Zn-Pb (0.242). While As-Cr (0.389) displayed a significant positive correlation at P < 0.05. In addition, Cr-Zn (0.008) and Ni-Pb (0.170) showed a relatively weak correlation (Table 5). Heavy metals having significant correlations did not mean they originated from the same source, which would depend upon the source and pathway between inter-elements correlation [57,76].

PCA was applied to obtain the validity of the source identification of metals through Kaiser-Meyer-Olkin, and is significant, according to the Bartlett’s test. The finding of the PCA variation diagram in rotated space, the total variance of “three rotated principal components” (PC) is 66.92% with eigenvalues >1. As and Cr were a heavy fall in PC1 with a total variance of 29.898%, having an eigenvalue of 2.093. Pb was identified as high loading with 20.203% of total variance as PC2 (Figure S2). Ni (0.741) and Zn (0.810) were strongly correlated with a total variance of 16.725% as PC3 (Table 6).

## 4. Discussion

The average concentration of Cr, Ni, Cu, Zn, and Pb is higher than the value in the “Weihe River basin” [77]. Cr concentration is a consequence of straight discharging and unprocessed waste from different textile industries and tanneries [78]. Cr exists in several valence states from −2 to +6, among which “0 (elemental metal), +3 (trivalent), and +6 (hexavalent)” are the most stable states. The health effect of Cr is related to the valence state of metal at the time of exposure. Biologically trivalent and hexavalent are considered to be the most important, where trivalent is an essential nutritional mineral [79]. Arsenic is regarded as toxic to humans as well as to aquatic organisms [10].

Excessive concentration of As can be connected to anthropogenic activities, for example, fertilizer used for agriculture, arsenical pesticides, copper arsenate treatment of wood, as well as tanning with certain chemicals, more likely arsenic sulfide [8,80]. Cu and Zn are important micronutrients for aquatic organisms, but toxic at high levels [10]. In sediments, metals were linked to their nearby traffic activities, i.e., copper used in car lubricant, chromium in alloy steel for auto parts, and stainless steel [8].

Overall, the concentration of metals in the studied area was relatively in between as compared with other rivers in China and the world. The concentration of As, Cr, and Ni in the Weihe River is greater than in the Luanhe River, China; and Cu, Cr, and Zn are less when compared to the Yangtze River, China. The Yellow River, Zijiang River, Hunan, and the Weihe River have nearly the same concentration of these metals (Table 4). Earlier studies in the Shaanxi basin clarified that sediments were mainly polluted by Cd in the Weihe River [1,77]. Major contents of metals were generated by an anthropogenic effect [81].

The Igeo was used to measure the different pollution levels in sediments. According to our results, most of the sites in the Weihe River were uncontaminated (class 0) because Igeo values were less than zero. Through all heavy metals, As has maximum accumulation at D3 and D14, which indicates that the sediments at these locations are moderately polluted by As and belong to class 2. Additionally, at few stations, the Igeo concentrations for the Cr, Cu, Zn, and Pb are greater than zero, which shows the minimal presence of Cr, Cu, Zn, and Pb, and places in class 1 “Uncontaminated to moderately contaminate”. However, the average values of Igeo for Cr, Cu, and Zn are less than zero. Moreover, arsenic had several positive values (greater than 0), which specify that the sediments are moderately contaminated by arsenic.

EF can be used to distinguish between sources of the element, which may be anthropogenic or natural. The sediments that have EF value between 0 and 1.5 suggest that their origin is natural or derived from crustal material. On the other hand, EF>1.5 indicates that these originated through anthropogenic activities. If the EF value is higher than 10, then these metals were considered non-crusted sources [82]. The average EF value for As was higher than 1.5, which suggests an anthropogenic effect on metals. The average EF values for Cr, Ni, Cu, Zn, and Pb were less than 1.5, which indicates the crustal or natural origin. Cr at site D7 and D8, Cu at D1, D7, and D13, Zn D11 and D13 and Pb at sites D2, D7, D8, D9, and D13, have EF values more than 1.5, which shows that the origin of these metals at these sites are most probably anthropogenic [82]. EF values below 5.0 will not be regarded significant, for the reason that such minor enrichments may result from differences within the composition of neighborhood soil materials along with reference sediment utilized in EF calculations [83].

Contamination factor results show that the Weihe River is moderately contaminated by As, Cr, Cu, Zn, Mn, and Pb, with a low contamination by Ni. As has a maximum value of CF at sites D3 and D14, indicating these sites being considerably contaminated, whereas D11 showed moderate contamination.

Potential ecological risk results elucidated that the highest ecological risk (ER) is for arsenic, and the lowest risk is for manganese. The ecological risk values of all metals were below 30, suggesting a slight pollution level. Only As at D3 had an ER value of more than 30, which is not a severe threat for ecology. In general, all measured metals had low ecological risk across all stations. Regarding risk index, As is the major contributor, and the other metals, Ni, Cr, Cu, Zn, Pb, and Mn exhibited low potential ecological risk indices. According to the risk index, sampling site D3 has a maximum risk index, and site D12 shows a minimum risk index. Our study showed the PER < 40 and RI < 110 for the Weihe River, which is solid evidence of low risk of these metals in the subjective area.

The principal component analysis was performed to compare the pattern between the heavy metals. PCA of the whole data set showed three PCs with eigenvalues >0.6 that illuminated about 66.92%. The first component accumulated for 29.89%, correlated (loading >0.6) with As and Cr, indicating the similar distribution patterns. While, the second component accumulated for 20.20%, correlated with Pb. However, Pb is the only element in the second component, which had a large load and measurement among all the other elements, and the concentration of this element is higher than background values. The third component of 16.72%, and correlated (loading >0.6) with Ni and Zn by showing high concentrations and primarily distributed in the sediments. Pearson correlation analysis indicates that As had a strongly positive significant correlation with Cr, Cu, and Mn, which revealed they were of the same source. However, Cr was negatively correlated with Ni, and Zn, which demonstrated that these metals could be from different sources. Similarly, Cu and Pb had a significant negative relationship with Mn, indicating the pair to have originated from different sources. Although these heavy metals have no severe risk, measures should be taken to stop heavy metals pollution in the studied area.

## 5. Conclusions

Sediment samples from fourteen sites have been taken, and heavy metals in the samples were ranked as follows: “Manganese (Mn) > chromium (Cr) > zinc (Zn) > copper (Cu) > nickel (Ni) > arsenic (As) > lead (Pb)”. To measure the contamination levels in the Weihe River, “geo accumulation index, enrichment factor and contamination factor” have been utilized. Further, the potential ecological risk and risk index have been calculated to evaluate the ecological risk of heavy metals. According to the geo-accumulation index, As belonged to class 1 (uncontaminated to moderate contamination), while Cr, Ni, Cu, Zn, Pb, and Mn belonged to class 0 (uncontaminated). According to the enrichment factor, As was originated through anthropogenic activities, and the Cr, Ni, Cu, Zn, and Pb were from a natural source. The potential ecological risk and total risk index were less than 40 and 110, respectively, which indicates that these heavy metals have low ecological risk. In the risk index, As showed the highest contribution at 53.43%, and Cr, Ni, Cu, Zn, Pb, and Mn were 5.82%, 7.26%, 13.86%, 2.59%, 14.55%, and 2.49%, respectively. According to the correlation matrix, a significant positive correlation existed among the following pairs: (As, Cr), (As, Mn), and (Ni, Zn), while relatively weak positive correlation has been found within pairs (Cr, Zn) and (Ni, Pb). Lastly, a negative correlation existed among (Cr, Ni), (Cu, Mn), and (Pb, Mn).

## Figures and Tables

**Table 1:** Geo-accumulation index (Igeo) and Enrichment factor (EF) classification.

Igeo		EF	
Igeo Classes	Sediment quality	EF Class	Sediment quality
Igeo ≤0	No pollution	EF<1	No pollution
Igeo=0–1	No to moderate pollution	EF<2	Very small pollution
Igeo=1–2	Moderate pollution	2<EF<5	Deficiency to small pollution
Igeo=2–3	Moderate to heavy pollution	EF=5–10	Moderate to high pollution
Igeo=3–4	Heavy pollution	EF=10–25	High pollution
Igeo=4–5	Heavy to extreme pollution	EF=25–50	Very high pollution
Igeo ≥5	Extreme pollution	EF>50	Exceptionally high pollution

**Table 2:** Ecological risk and risk index (RI) classification.

ER Level	Value of ER	Risk	Value of RI	Risk
0	ER<40	Low	RI<110	Low
1	ER=40–80	Moderate	RI=110–200	Moderate
2	ER=80–160	Considerable	RI=200–400	Considerable
3	ER=160–320	High	RI≥400	Very High
4	ER≥320	Very High		

**Table 3:** Variation in concentrations of heavy metal in the sediment from different stations of the Weihe River.

Location	As	Cr	Ni	Cu	Zn	Pb	Mn
D1	27.55	84.64	38.76	69.34	75.84	16.72	716.34
D2	31.98	60.54	42.83	55.77	133.27	35.42	918.44
D3	39.93	122.66	39.28	52.63	108.65	25.94	1036.63
D4	22.89	108.79	37.28	57.44	82.98	24.25	1212.79
D5	35.43	142.93	22.98	18.23	95.75	17.46	1152.61
D6	29.38	104.74	32.63	35.53	111.45	17.68	842.41
D7	26.62	138.67	15.43	68.40	104.27	27.57	519.25
D8	35.98	117.87	46.68	58.47	95.64	26.49	738.43
D9	23.69	93.78	39.98	35.39	71.32	33.30	686.94
D10	26.88	108.67	46.24	62.43	78.74	21.63	1088.73
D11	18.43	105.67	62.38	53.45	141.83	19.75	828.73
D12	20.67	98.46	54.94	32.30	88.87	23.99	940.64
D13	29.59	113.87	57.46	65.94	143.64	36.39	718.64
D14	39.24	138.37	43.76	67.93	116.28	15.62	1035.43
Minimum	18.43	60.54	15.43	18.23	71.32	15.62	519.25
Maximum	39.93	142.93	62.38	69.34	143.64	36.39	1212.79
Average	29.16	109.98	41.47	52.37	103.47	24.44	888.29

**Table 4:** The concentration of metals in the Weihe River compared with different rivers of the world from literature (mg/kg).

River	Cu	As	Cr	Ni	Zn	Mn	Pb	Reference
Weihe River, Xian, China	18.23–69.34	18.43–39.93	60.54–142.93	15.43–62.38	71.32–143.64	519.25–1212.79	15.62–36.39	This Study
Zijiang River, Hunan, China	18.37–59.01	6.90–74.34	48.47–95.32	21.50–52.29	42.41–251.61	570.75–2106.73	12.70–104.32	[65]
Yangtze River, China	129	29.90	205	NA	1142	NA	98	[66]
Jialu River, China	8.82–107.61	2.39–14.57	40.04–96.39	19.75–80.26	42.39–210.00	NA	14.79–51.17	[64]
Luanhe River, China	NA	3.4–13.5	9.6–35.6	3.5–35.8	NA	NA	22.6–43.7	[63]
Yellow River, China	30–102	14–48	41–128	NA	NA	NA	26–78	[67]
Korotoa River, Bangladesh	76	25	109	95	NA	NA	58	[68]
Axios River, Greece	93	40	180	188	271	NA	140	[60]
River Po, Italy	90.1	NA	NA	16198.5	645	NA	98.5	[61]
Gomti River, India	245.33	NA	88.7	76.08	343.47	834.7	156.2	[69]
Chenab River, Pakistan	5.80–9.40	NA	NA	NA	11.7–50.5	245–851	2.4–32.4	[59]
Almendares River, Cuba	420.8	NA	23.4	NA	708.8	NA	189	[70]
Nile River Egypt	81	NA	274	112	221	2810	23.2	[71]
South Platte River, USA	480	31	71	NA	3700	6700	270	[72]
Tees River, UK	76.9	NA	NA	NA	1920	5240	6880	[62]

NA represents “Not Available”.

**Table 5:** Pearson correlation analysis results of metals in sediments.

	As	Cr	Ni	Cu	Zn	Pb	Mn
As	1						
Cr	0.389 *	1					
Ni	−0.289	−0.380 **	1				
Cu	0.092 **	−0.032	0.157	1			
Zn	0.146	0.008 *	0.361 **	0.203 **	1		
Pb	−0.083	−0.367	0.170 *	0.120	0.242 **	1	
Mn	0.214 **	0.132	0.046	−0.286 *	−0.132	−0.365 **	1

* Significant correlation at *p* < 0.05. ** Significant correlation at *p* < 0.01.

**Table 6:** Values of rotated component analysis of metals in the Weihe River sediment.

Metals	Components		
	1	2	3
As	**0.805**	−0.049	0.164
Cr	**0.788**	−0.157	−0.153
Ni	−0.510	−0.112	**0.741**
Cu	0.205	0.551	0.352
Zn	0.164	0.218	**0.810**
Pb	−0.308	**0.623**	0.213
Mn	0.126	−0.868	0.140
Eigenvalue	2.093	1.421	1.171
% Total variance	29.898	20.203	16.725
Cumulative % variance	29.898	50.201	66.926

PCA values > 0.6 are presented in bold.

## Supplementary Materials

The following are available online at https://www.mdpi.com/1660-4601/17/3/1070/s1, **Figure S1**: variation in concentrations of heavy metal in the sediment collected from different stations of the Weihe River. Figure S2: three principal components plot in the principal component analysis (PCA). Table S1: background concentration of heavy metals used in this study. Table S2: variation in contamination levels “geo accumulation index (Igeo), enrichment factor (EF), contamination factor (CF), ecological risk (ER), and risk index (RI)” in the Weihe River.
