# Supplementary material for: Contamination Level, Ecological Risk, and Source Identification of Heavy Metals in the Hyporheic Zone of the Weihe River, China
Source: Int J Environ Res Public Health. 2020 Feb 7;17(3):1070. doi: 10.3390/ijerph17031070 (PMC7037357; doi:10.3390/ijerph17031070)
Supplement: Supplementary file 1 [file ijerph-17-01070-s001.pdf]

Contamination Level, Ecological Risk and Source Identification of Heavy Metals in the Hyporheic Zone of the Weihe River, China

Muhammad Irfan Ahamad, Jinxi Song *, Haotian Sun, Xinxin Wang, Muhammad Sajid Mehmood, Muhammad Sajid, Ping Su and Asif Jamal Khan

Submitted to: International Journal of Environmental Research and Public Health

Supplementary material

The detailed procedure for total metal digestion：

In brief, a mass of 0.25 g dry sediment sample was weighed into a set of 10 mL Teflon vessel. About 2 mL concentrated HNO_3 and 2 mL concentrated HCL and 1 mL concentrated H_2O_2 were added to the samples and the vessel were left on a hot plate for one day to remove organics. Afterwards, the samples were dried at 120 °C and the residues were dissolved in 1 mL concentrated HNO_3 and 2 mL concentrated HF and subjected to ultrasonic treatment for 20 min. Then the samples were put into sealed vessel and placed in an oven at 180 °C for 24 hour. This procedure generated a clear solution from the samples. After evaporation at 120 °C, the samples were subjected to ultrasound treatment for another 30 min and dissolved in 1% HNO_3. Then sample cool down at room temperature and prepared 50% volumetric flask. ICP-MS was used to determine total concentrations of trace metals in the sediments [1].

"Gao, L.; Gao, B.; Xu, D.; Peng, W.; Lu, J. Multiple assessments of trace metals in sediments and their response to the water level fluctuation in the Three Gorges Reservoir, China. *Sci. Total Environ.* **2019**, *648*, 197–205."

No of Figures: 2

Figure S1. Variation in concentrations of heavy metal in the sediment collected from different stations of the Weihe River

Figure S2. Three principal components plot in the principal component analysis (PCA)

No of Tables: 2

Table S1. Background concentration of heavy metals used in this study

Table S2. Variation in contamination levels "geo accumulation index (Igeo), enrichment factor (EF), contamination factor (CF), ecological risk (ER), pollution load index (PLI) and risk index (RI)" in the Weihe River.

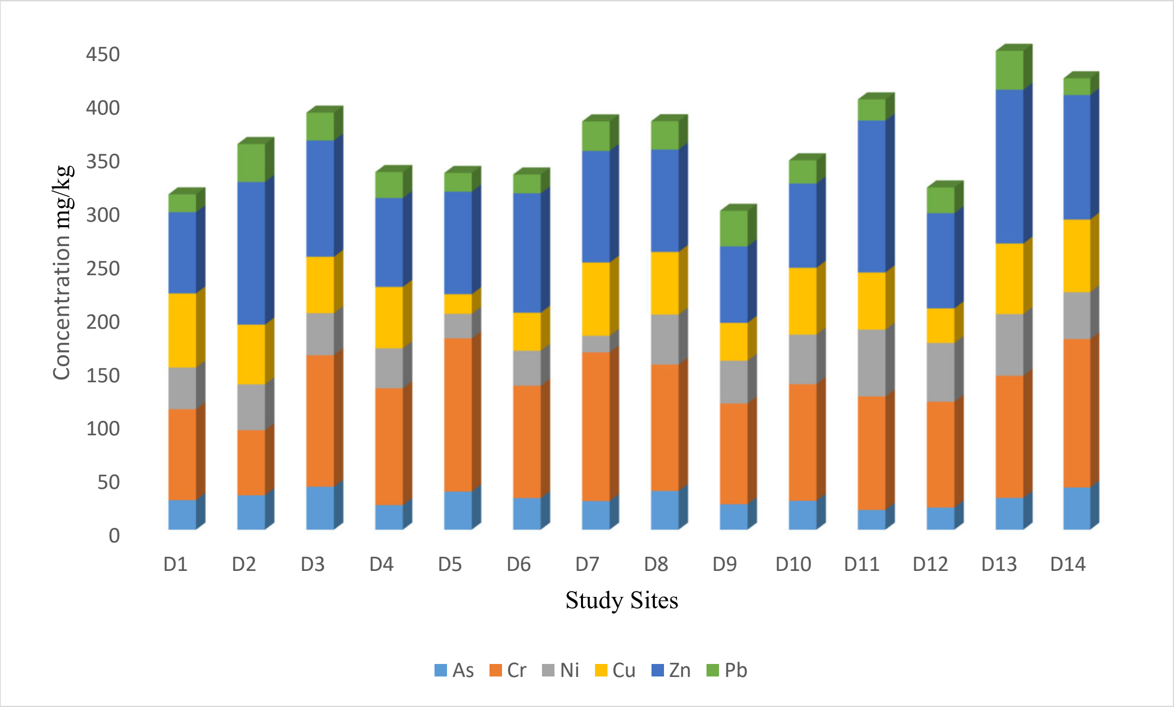

Figure S1. Variation in concentrations of heavy metal in the sediment collected from different stations of the Weihe River

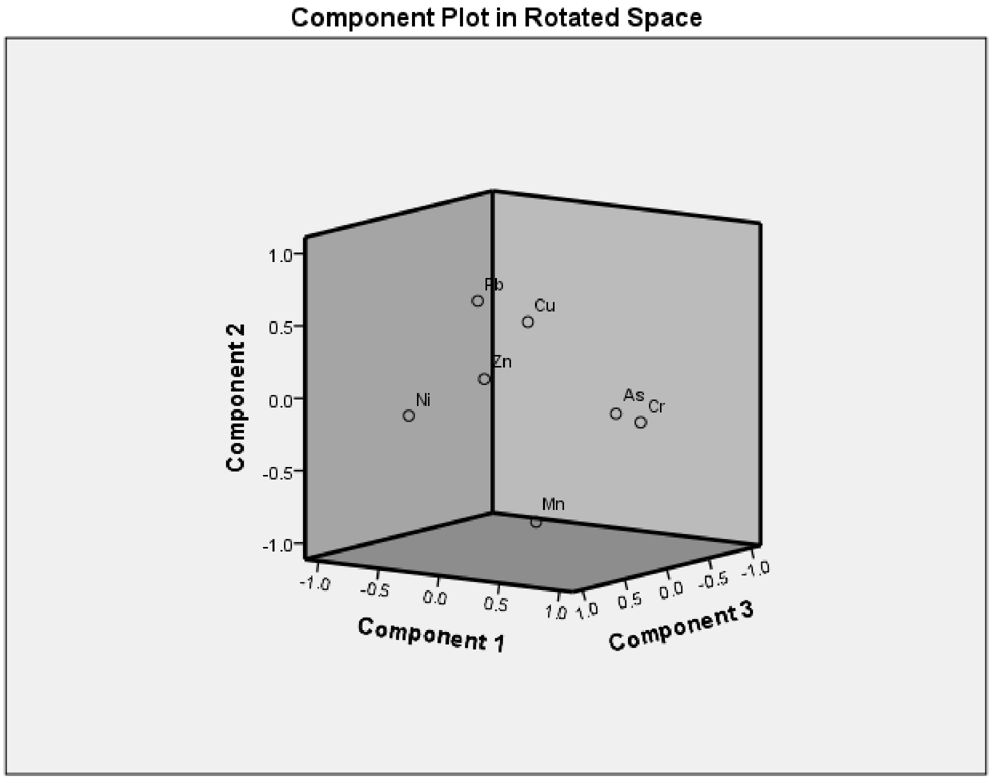

Figure: S2.Three principal components plot in the principal component analysis (PCA)

Table S1. Background concentration of heavy metals used in this study

Metal	Background Value	Metal	Background Value	Metal	Background Value
As	13	Cr	90	Ni	68
Cu	45	Zn	95	Pb	20
Mn	850				

Table S2. Variation in contamination levels "geo accumulation index (Igeo), enrichment factor (EF), contamination factor (CF), ecological risk (ER) and risk index (RI)" in the Weihe River.

Sites	As				Cr				Ni				Cu				Zn				Pb				Mn			RI
	Igeo	EF	CF	ER	Igeo	EF	CF	ER	Igeo	EF	CF	ER	Igeo	EF	CF	ER	Igeo	EF	CF	ER	Igeo	EF	CF	ER	Igeo	CF	ER	
D1	0.50	2.51	2.12	21.19	-0.67	1.12	0.94	1.88	-1.40	0.68	0.57	2.85	0.04	1.83	1.54	7.70	-0.91	0.95	0.80	0.80	-0.84	0.99	0.84	4.18	-0.83	0.84	0.84	39.44
D2	0.71	2.28	2.46	24.60	-1.16	0.62	0.67	1.35	-1.25	0.58	0.63	3.15	-0.28	1.15	1.24	6.20	-0.10	1.30	1.40	1.40	0.24	1.64	1.77	8.86	-0.47	1.08	1.08	46.63
D3	1.03	2.52	3.07	30.71	-0.14	1.12	1.36	2.73	-1.38	0.47	0.58	2.89	-0.36	0.96	1.17	5.85	-0.39	0.94	1.14	1.14	-0.21	1.06	1.30	6.48	-0.30	1.22	1.22	51.02
D4	0.23	1.23	1.76	17.61	-0.31	0.85	1.21	2.42	-1.45	0.38	0.55	2.74	-0.23	0.89	1.28	6.38	-0.78	0.61	0.87	0.87	-0.31	0.85	1.21	6.06	-0.07	1.43	1.43	37.51
D5	0.86	2.01	2.73	27.25	0.08	1.17	1.59	3.18	-2.15	0.25	0.34	1.69	-1.89	0.30	0.41	2.03	-0.57	0.74	1.01	1.01	-0.78	0.64	0.87	4.36	-0.15	1.36	1.36	40.87
D6	0.59	2.28	2.26	22.60	-0.37	1.17	1.16	2.33	-1.64	0.48	0.48	2.40	-0.93	0.80	0.79	3.95	-0.35	1.18	1.17	1.17	-0.76	0.89	0.88	4.42	-0.60	0.99	0.99	37.86
D7	0.45	3.35	2.05	20.47	0.04	2.52	1.54	3.08	-2.72	0.37	0.23	1.13	0.02	2.49	1.52	7.60	-0.45	1.80	1.10	1.10	-0.12	2.26	1.38	6.89	-1.30	0.61	0.61	40.89
D8	0.88	3.19	2.77	27.68	-0.20	1.51	1.31	2.62	-1.13	0.79	0.69	3.43	-0.21	1.50	1.30	6.50	-0.58	1.16	1.01	1.01	-0.18	1.52	1.32	6.62	-0.79	0.87	0.87	48.72
D9	0.28	2.26	1.82	18.23	-0.53	1.29	1.04	2.08	-1.35	0.73	0.59	2.94	-0.93	0.97	0.79	3.93	-1.00	0.93	0.75	0.75	0.15	2.06	1.66	8.32	-0.89	0.81	0.81	37.06
D10	0.46	1.61	2.07	20.67	-0.31	0.94	1.21	2.41	-1.14	0.53	0.68	3.40	-0.11	1.08	1.39	6.94	-0.86	0.65	0.83	0.83	-0.47	0.84	1.08	5.41	-0.23	1.28	1.28	40.94
D11	-0.08	1.45	1.42	14.18	-0.35	1.20	1.17	2.35	-0.71	0.94	0.92	4.59	-0.34	1.22	1.19	5.94	-0.01	1.53	1.49	1.49	-0.60	1.01	0.99	4.94	-0.62	0.97	0.97	34.45
D12	0.08	1.44	1.59	15.90	-0.46	0.99	1.09	2.19	-0.89	0.73	0.81	4.04	-1.06	0.65	0.72	3.59	-0.68	0.85	0.94	0.94	-0.32	1.08	1.20	6.00	-0.44	1.11	1.11	33.76
D13	0.60	2.69	2.28	22.76	-0.25	1.50	1.27	2.53	-0.83	1.00	0.85	4.23	-0.03	1.73	1.47	7.33	0.01	1.79	1.51	1.51	0.28	2.15	1.82	9.10	-0.83	0.85	0.85	48.30
D14	1.01	2.48	3.02	30.19	0.04	1.26	1.54	3.07	-1.22	0.53	0.64	3.22	0.01	1.24	1.51	7.55	-0.29	1.00	1.22	1.22	-0.94	0.64	0.78	3.91	-0.30	1.22	1.22	50.37
Minimum	-0.08	1.23	1.42	14.18	-1.16	0.62	0.67	1.35	-2.72	0.25	0.23	1.13	-1.89	0.30	0.41	2.03	-1.00	0.61	0.75	0.75	-0.94	0.64	0.78	3.91	-1.30	0.61	0.61	33.76
Maximum	1.03	3.35	3.07	30.71	0.08	2.52	1.59	3.18	-0.71	1.00	0.92	4.59	0.04	2.49	1.54	7.70	0.01	1.80	1.51	1.51	0.28	2.26	1.82	9.10	-0.07	1.43	1.43	51.02
Average	0.54	2.24	2.24	22.43	-0.33	1.23	1.22	2.44	-1.38	0.60	0.61	3.05	-0.45	1.20	1.16	5.82	-0.50	1.10	1.09	1.09	-0.35	1.26	1.22	6.11	-0.56	1.05	1.05	41.99
